# The Role of Clinical and Ultrasound Enthesitis Scores in Ankylosing Spondylitis

**DOI:** 10.3390/life11030218

**Published:** 2021-03-09

**Authors:** Alesandra Florescu, Vlad Pădureanu, Dan Nicolae Florescu, Anca Bobircă, Lucian-Mihai Florescu, Ana-Maria Bumbea, Rodica Pădureanu, Anca Emanuela Mușetescu

**Affiliations:** 1 Department of Rheumatology, Emergency Clinical County Hospital of Craiova, 200642 Craiova, Romania; alesandracioroianu@yahoo.com; 2 Department of Internal Medicine, University of Medicine and Pharmacy of Craiova, 200349 Craiova, Romania; vldpadureanu@yahoo.com; 3 Department of Gastroenterology, University of Medicine and Pharmacy of Craiova, 200349 Craiova, Romania; nicku.dan@gmail.com; 4 Department of Rheumatology, Carol Davila University of Medicine and Pharmacy, 050474 Bucharest, Romania; ancu_boca@yahoo.com; 5 Department of Radiology and Medical Imaging, University of Medicine and Pharmacy of Craiova, 200349 Craiova, Romania; floresculm@gmail.com; 6 Department of Rehabilitation Medicine, University of Medicine and Pharmacy of Craiova, 200349 Craiova, Romania; anamariabumbea@yahoo.com; 7 Department of Internal Medicine, Emergency Clinical County Hospital of Craiova, 200642 Craiova, Romania; 8 Department of Rheumatology, University of Medicine and Pharmacy of Craiova, 200349 Craiova, Romania; anca_musetescu@yahoo.com

**Keywords:** enthesitis, musculoskeletal ultrasound, SPARCC, BUSES

## Abstract

Introduction: Ankylosing spondylitis (AS) is a chronic inflammatory disease, part of the spondyloarthritis (SpA) group, characterized by axial (spine and sacroiliac joints), entheseal, and peripheral joint involvement, which is frequently associated with extra-articular manifestations. Material and Methods: The study included a number of 30 patients diagnosed with AS according to the New York modified criteria, with history of entheseal pain, hospitalized between 2016–2018 in the Department of Rheumatology of the Emergency County Hospital of Craiova. Results: Regarding the Belgrade Ultrasound Enthesitis Score (BUSES) score and the disease activity calculated using the Ankylosing Spondylitis Disease Activity Score (ASDAS), they did not show a statistically significant association (*p* = 0.738). Additionally, BUSES did not have a statistically significant association with the disease activity quantified by the Bath Ankylosing Spondylitis Disease Activity Index (BASDAI) score (*p* = 0.094). The Spondyloarthritis Research Consortium of Canada Enthesitis Index (SPARCC) clinical score was not statistically associated with ASDAS (*p* = 0.434) nor with BASDAI (*p* = 0.130). The SPARCC clinical score and the BUSES ultrasound score were statistically significantly associated, registering a value of *p* = 0.018. Conclusions: Our study proved a significant correlation between SPARCC and BUSES, although in literature the evidence is contrasting.

## 1. Introduction

Ankylosing spondylitis (AS) is a chronic inflammatory disease, part of the spondyloarthritis (SpA) group, characterized by axial (spine and sacroiliac joints), entheseal, and peripheral joint involvement, which is frequently associated with extra-articular manifestations in the presence of human leukocyte (HLA) -B27 antigen [1,2].

Scapulohumeral and coxofemoral joints are the most commonly involved of the peripheral joints, affected in 35% of patients with AS. Impairment of the hip, through repeated coxitis episodes, with the onset of secondary hip osteoarthritis, may lead to an increased disability of the patient with AS. More rarely, the oligoarticular, asymmetrical type, predominantly in the lower limbs, can be highlighted [3,4,5].

The enthesis is the insertion of a tendon, ligament, capsule, or fascia on the bone. Entheseal involvement, defined as enthesitis, is considered the characteristic hallmark of AS. Most commonly, the entheses of the lower limbs are involved, especially the insertion of the Achilles tendon or the plantar fascia on the calcaneus, but also the ligamentous structures of the intervertebral disc, the entheses at the level of the spinal processes, the manubrio-sternal joints, the pubic symphysis, the iliac crest, the trochanters, or the patella [6,7]. The defining enthesitic involvement in AS starting from the morphological, functional, and pathogenic characteristics of the enthesis or the concept of the enthesis organ has a series of particularities that make it difficult to evaluate.

Of great importance in current practice are expression of enthesitis by pain and sensitivity to palpation frequently in the absence of local swelling, which makes it difficult to recognize; the swelling characteristic of large insertions in the lower limbs and of lesser extent in the upper limbs; juxta-articular position of entheses that may lead to confounding with articular pain, and often the presence of entheseal inflammatory changes in the absence of increased inflammatory markers, the enthesis being a relatively avascular structure.

The concept of enthesis organ or synovio-entheseal complex represented by enthesis, fibrocartilage, subchondral bone, and bursa, with synovial membrane and fatty tissue, explains the origin and sequence of inflammatory events in fibrocartilaginous entheses, with the Achilles tendon as prototype, starting from the proinflammatory potential of the perientheseal fatty tissue located in the epitendon and endotendon, in the angle of entheseal insertion and bursa, richly vascularized and innervated, capable of releasing proinflammatory cytokines and growth factors through endocrine and paracrine potential. 

The intimate anatomical and functional link between the synovial membrane and the enthesis explains the impact of mechanical stress and microlesions at the entheseal level in promoting synovial inflammation, recruiting the potent proinflammatory cell arsenal of macrophages and lymphocytes at the deposition site of microbacterial arthritogenic peptides in the entheseal insertion to genetically susceptible individuals.

The measurement of enthesitis in clinical practice is done using scores validated especially for AS, such as Mander Enthesis Index (MEI), Maastricht Ankylosing Spondylitis Enthesis (MASES), MAJOR enthesitis index, and Spondyloarthritis Research Consortium of Canada Enthesitis Index (SPARCC) [8]. 

In order to detect the subclinical enthesitis and to evaluate the response to the treatment, musculoskeletal ultrasound (MUS) scores were developed for the evaluation of entheses in AS such as Glasgow Ultrasound Enthesitis Index (GUESS), D’Agostino, Spanish Sonographic Enthesitic Index (SEI), Madrid Sonographic Enthesitis Index (MASEI), and Belgrade Ultrasound Enthesitis Score (BUSES) [9]. 

There are many methods of evaluating enthesitis, in particular clinical scores and in the latter years several ultrasound scores, but a highly accurate technique that includes symptomatology, structural lesions, and inflammatory markers at different entheseal sites is lacking as well as definite correlations between these enthesis-related disease panels.

## 2. Materials and Methods

### 2.1. Patients 

The study included a number of 30 patients diagnosed with AS according to the New York modified and ESSG (European Spondyloarthropathy Study Group) criteria [10], with history of entheseal pain, hospitalized between 2018–2020 in the Department of Rheumatology of the Emergency County Hospital of Craiova. All patients expressed their agreement to be a part of this study. The study was approved by local institutional ethics committees of the University of Medicine and Pharmacy of Craiova (Registration No. 16) according to European Union Guidelines (Declaration of Helsinki).

### 2.2. Demographic Characteristics and The Assessment of Clinical and Laboratory Data

All patients underwent clinical examination, laboratory tests, and MUS. The laboratory tests consisted of complete blood count (CBC), liver enzymes, serum creatinine, erythrocyte sedimentation rate (ESR) with a normal value <10 mm/h, and C reactive protein (CRP) with a normal value <5 mg/L. 

Entheseal involvement was determined using both a clinical score (SPARCC) and an echographic one (BUSES). 

Using the SPARCC clinical score we quantified the absence (0) or the presence (1) of pain upon palpation of the insertion of 8 bilateral entheses, as follows: the medial and lateral humeral epicondyles, the insertion of the tendon of the supraspinatus muscle on the great humeral tuberosity, the great femoral trochanter, the insertion of the quadriceps tendon on the superior pole of the patella, the insertion of the patellar ligament on the inferior pole of the patella or on the tibial tubercle, the insertion of the Achilles tendon on the calcaneus, and the insertion of the plantar fascia on the calcaneus. 

Ultrasound (US) examinations were performed on an Esaote MyLabX6 US system, using a multifrequency probe of 6–18 MHz. 

Grey-scale and power Doppler (PD) techniques in both longitudinal and transverse planes were used in order to assess the presence of increased thickness of the enthesis, hypoechogenicity, lack of normal fibrillary aspect, enthesophtytes, erosions, and power Doppler signal at the enthesis. 

According to BUSES, we evaluated 12 entheses (plantar fascia, Achilles tendon, proximal and distal patellar tendon, quadriceps tendon, and common extensor tendon on the lateral epicondyle), both in gray-scale and power Doppler, quantifying tendon thickness (0 or 1), hypoechogenicity with loss of fibrillar pattern (0 or 1), the presence of enthesophytes (0 or 1), erosions (0 or 4), and power Doppler signal (0 or 4).

Standardized scores such as Ankylosing Spondylitis Disease Activity Score (ASDAS), Bath Ankylosing Spondylitis Disease Activity Index (BASDAI) were used in order to assess disease activity in the group of patients. 

### 2.3. Statistical Analysis

Statistical analyses of the data were performed using Graph Pad Prism 9 software. The relationship between variables was analyzed using unpaired t test. Values less than 0.05 for *p* were considered statistically significant. Summary statistics of the mean ± standard deviation (SD) are presented for continuous variables. 

## 3. Results

The study included 30 patients with AS: 23 males and 7 females with mean age of 38.36 ± 10.87 years. 

**Figure 1:**
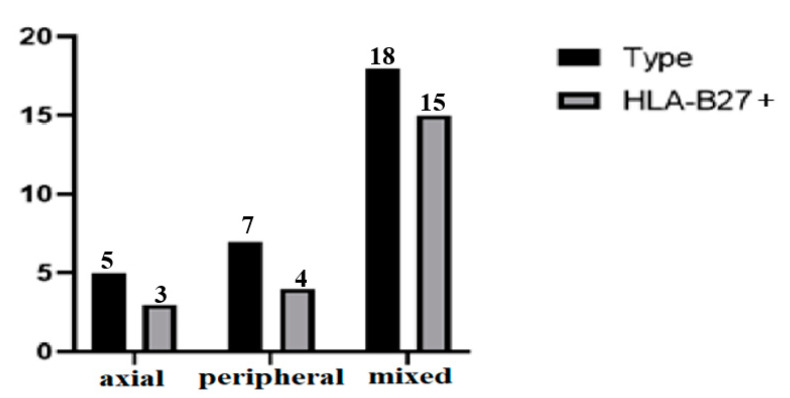
Positivity of HLA-B27 according to the type of articular involvement.

The patients had either the axial form of AS, but also with entheseal involvement (17%); the peripheral form of AS (23%); or mixed form of AS which included both axial and peripheral joint involvement (60%). HLA-B27 was positive in 73.33% of the patients. The positivity of HLA-B27 was evaluated in the study group, according to the form of the disease, as follows: 60% of the patients with axial form, and 57% and 83% of the patients with peripheral and mixed forms, respectively (Figure 1). 

Disease activity calculated using ASDAS-CRP was within the moderate range in 10% of patients, within the high range in 7% of cases, and within the very high range in 83% of patients. 

The baseline characteristics of the patients enrolled in the study are highlighted in Table 1. 

Out of the 480 examined tendons included in the SPARCC score, 150 were sensitive at palpation. The affected entheses were distributed as follows: medial humeral epicondyles (4%), lateral humeral epicondyles (4.66%), greater humeral tuberosity (4.66%), greater femoral trochanter (15.33%), superior patellar pole (13.33%), inferior patellar pole (6%), Achilles tendon (28%), and plantar fascia (24%). 

**Figure 2:**
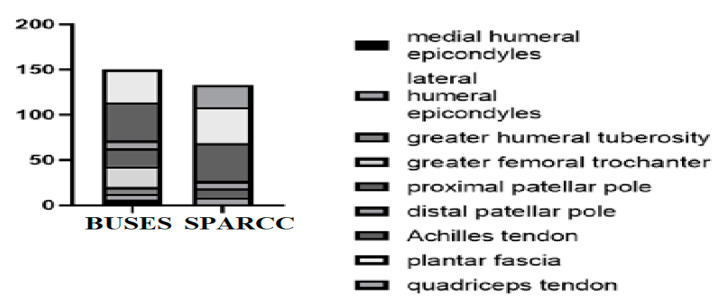
Distribution of affected tendons according to the Belgrade Ultrasound Enthesitis Score (BUSES) and the Spondyloarthritis Research Consortium of Canada Enthesitis Index (SPARCC).

**Figure 3:**
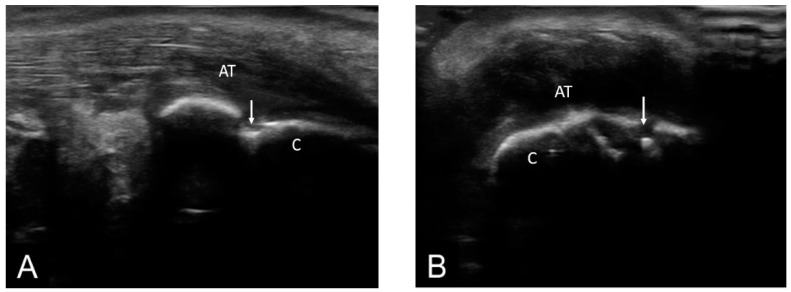
AT—Achilles tendon; C—calcaneus; ↓—calcaneus erosion; pathology—longitudinal (**A**) and transverse scan (**B**) in grey-scale of the Achilles tendon showing erosions in two perpendicular planes.

**Figure 4:**
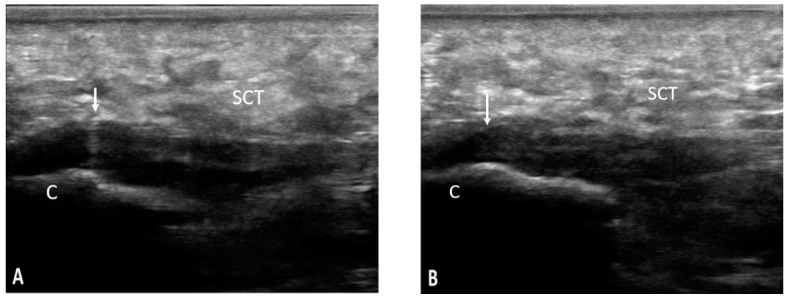
SCT—subcutaneous cellular tissue; C—calcaneus; ↓—plantar fascia; pathology—longitudinal scan of the plantar fascia bilaterally (**A**,**B**) showing a hypoechoic aspect, with loss of fibrillary pattern, thickening, and anterior convexity of the fascia with the presence of irregularities of the calcaneus cortex at the insertion.

According to BUSES, we evaluated 360 entheses out of which 133 presented ultrasonographic changes. The MUS signs of enthesitis were distributed as follows: plantar fascia (30%), Achilles tendon (31.57%), proximal patellar tendon (7.51%), distal patellar tendon (6.01%), quadriceps tendon (18.04%), and common extensor tendon on the lateral epicondyle (6.76%) (Figure 2, Figure 3 and Figure 4).

**Figure 5:**
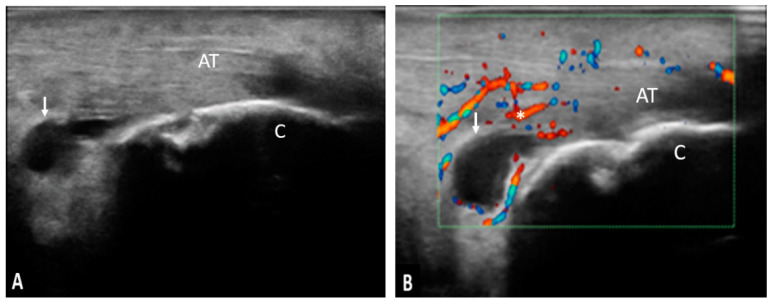
(**A**,**B**). AT—Achilles tendon; C—calcaneus;↓—retrocalcaneal bursa; *—power Doppler signal; pathology—longitudinal scan in grey-scale (**A**) and power Doppler (**B**) of the Achilles tendon showing a moderate effusion in the retrocalcaneal bursa and the presence of power Doppler signal.

The Achilles tendon was the most frequently involved in the inflammatory process both from the clinical and ultrasonographic point of view. Taking into account the characteristics evaluated by ultrasound, 42 of the examined Achilles tendons presented the following inflammatory changes: thickening (90.47%), hypoechogenicity (90.47%), loss of fibrillary pattern (90.47%), enthesophytes (76.19%), calcifications (66.66%), erosions (66.66%), power Doppler signal (28.57%), retrocalcaneal bursitis (28.57%), and retroachilles bursitis (23.80%) (Figure 5). 

ESR values did not show a statistically significant association with the SPARCC clinical score (*p* = 0.619) and with the BUSES ultrasound score (*p* = 0.200). The CRP values did not have a statistically significant association with the SPARCC clinical score (*p* = 0.137) and the BUSES ultrasound score (*p* = 0.102).

The BUSES ultrasound score and the disease activity calculated using ASDAS did not show a statistically significant association (*p* = 0.738). Additionally, BUSES did not have a statistically significant association with the disease activity quantified by the BASDAI score (*p* = 0.094).

The SPARCC clinical score was not statistically associated with ASDAS (*p* = 0.434) nor with BASDAI (*p* = 0.130).

**Figure 6:**
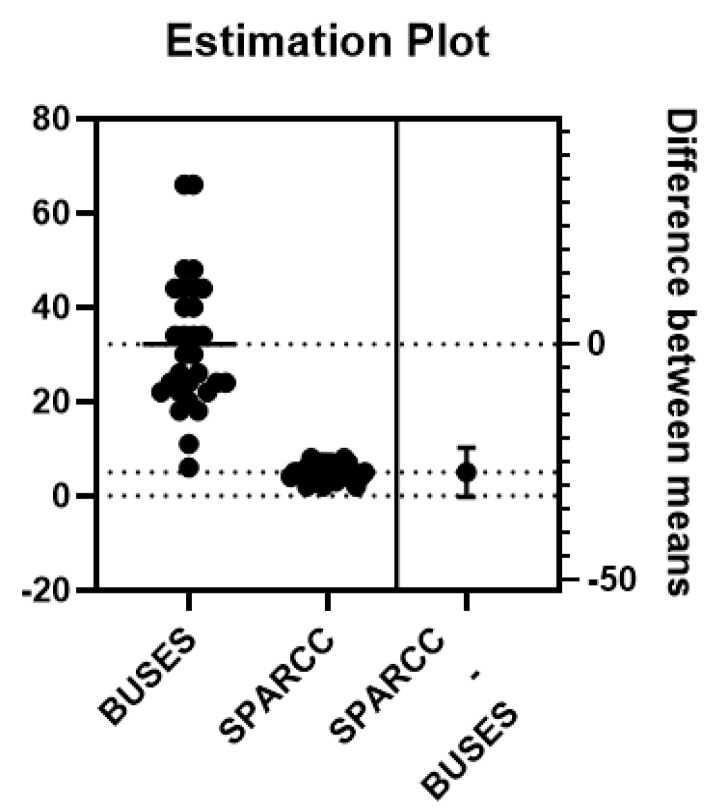
Estimation plot of t test between BUSES and SPARCC.

The SPARCC clinical score and the BUSES ultrasound score were statistically significantly associated, registering a value of *p* = 0.018 (Figure 6).

## 4. Discussion

Multiple studies in the literature have shown the superiority of MUS compared to clinical examinations in detecting inflammatory changes at the entheses level. The PD signal is a complementary MUS instrument used to increase sensitivity in detecting inflammation. Despite evidence supporting the use of PD to detect subclinical inflammation and to track treatment response of patients with SpA, there is a lack of standardized definitions of enthesitis and ultrasonographic parameters, which makes it difficult to compare data in various studies [11].

In a study by Alcade et al. (2007), who evaluated entheseal involvement using a score based only on the examination of lower limb entheses, the vast majority of patients presented enthesitis. The most frequent entheses involved in the inflammatory process were the Achilles tendon and the plantar fascia, results consistent with our study, although BUSES score evaluates more entheseal sites. The authors also pointed out that the ultrasound score did not correlate with the values of the inflammatory markers nor with the activity of the disease, results also found in our study [12,13].

The most common ultrasonographic changes in the Achilles tendon were increased tendon thickness, hypoechogenicity, and loss of fibrillary pattern (over 90%), results consistent with other studies in literature that showed the presence of these changes in over 50% of the patients included in the studies [14].

The presence of ultrasonographically detected bursitis was also studied, the most frequent bursae involved in the inflammatory process being the retrocalcaneal and the suprapatellar bursa. Similar results were obtained in the study conducted by Balint et al. (2002) [15].

SPARCC is a relatively new score that evaluates only peripheral entheses, which are more easily accessible for MUS evaluation. Thus, SPARCC can be successfully used to compare clinical and imaging data. Multiple recent studies have established that ultrasonography together with PD imaging is useful for the early diagnosis of enthesitis. Of the sonographic indices, the most used are the GUESS and SEI scores [16,17].

The ultrasound score did not correlate with BASDAI in our study. This could be due to the fact that BASDAI quantifies global disease activity and functional status, with only a few questions focusing on entheseal pain. The axial involvement in AS in our cohort included the entheseal involvement, but the study group included mainly patients with mixed articular involvement. BASDAI evaluates the overall disease activity and is not centered only on entheseal, peripheral, or axial involvement. Moreover, BASDAI is a subjective method of determining disease activity, the ASDAS score being a more reliable tool in assessing disease activity. In fact, not all tests used to quantify enthesitis measure the same characteristics; thus, there is a discrepancy between the scores of overall clinical activity and the disease activity at the entheseal level [18,19].

The SPARCC score did not show a statistically significant association with the markers of inflammation, ESR and CRP, a proven result in multiple studies in the literature [20].

A study by Kristensen et al. evaluated the association between SPARCC and the total enthesitis ultrasound score, demonstrating a moderate correlation between SPARCC and the ultrasound examination. From the point of view of the detected changes, the hypoechogenicity and the tendon thickness were the characteristics most strongly associated with SPARCC, while the presence of enthesophytes, erosions, and PD signal did not show a statistically significant correlation with SPARCC [21]. In our study, SPARCC and BUSES presented a significant association (*p* = 0.018).

In a study conducted by Hamdi et al, the SPARCC score showed a statistically significant correlation with the total sonographic score, a result which confirms the conclusions obtained in our study [22].

Regarding the association between the BUSES ultrasound score and the disease activity evaluated through ESR and CRP, we did not show a statistically significant correlation between BUSES and the inflammation markers, results also highlighted in a study by Balint et al. This may be due to the fact that, for the most part, the score includes the lower limb entheses, completely disregarding the general condition of the patients [23].

In our study, ASDAS did not show a statistically significant correlation with either BUSES or SPARCC. We believe that this finding is supported by the fact that ASDAS measures the global disease activity, not focusing on the pain or MUS abnormalities at the entheses level. A study conducted by Miguel et al. (2011) evaluated the grey-scale and power Doppler entheseal involvement of the Achilles tendon. They showed that there was a positive correlation between the ultrasound parameters, ESR, CRP, and ASDAS values even at 6 and 12 months from the initial evaluation. However, the ultrasound parameters were not associated with BASDAI values, a result concordant with our study. However, the study of the authors presented some limitations regarding the overall assessment of the entheseal involvement, since they only evaluated the Achilles tendon [24].

The study by Sivas et al. (2008) used the MASES score as a way of assessing enthesitis. The authors established that there were no correlations between the entheseal indices, the laboratory parameters, and the clinical activity of the disease. They also argue that ESR and CRP are used for acute phase assessment and do not always increase according to subjective symptoms, thus having poor correlations with clinical disease activity and radiological progression. Additionally, MASES showed a significant correlation with BASDAI, but not with BASFI (Bath Ankylosing Spondylitis Functional Index), in contrast to other studies described in the literature. This proves, once again, the inhomogeneity of the conclusions from the studies performed on patients with AS [25].

Studies in the literature have shown that there is no consensus regarding the evaluation of patients with AS. The inflammatory markers do not reflect the disease activity, nor the activity evaluated using clinical and ultrasound scores of the entheseal involvement. The BASDAI and BASFI questionnaires are subjective, having only one question that quantifies the enthesitis/enthesopathy, which leads to discrepancies between the indices and the entheseal evaluation. ASDAS evaluates the overall activity of the disease and does not quantify peripheral, axial, and entheseal activity separately. Thus, there is no gold standard regarding the evaluation of patients with AS [26].

## 5. Conclusions

Our study proved a significant correlation between SPARCC and BUSES, although in literature the evidence is contrasting. Further studies have to be conducted in order to debate the inconsistencies related to clinical and MUS examinations in patients with AS.

## Figures and Tables

**Table 1:** Baseline characteristics of the study group.

	Age	ESR (mm/h)	CRP (mg/L)	ASDAS	BASDAI	SPARCC	BUSES
**Mean**	38.36	44.63	21.90	4.51	7.46	5	32.23
**Standard Deviation**	10.87	22.88	21	1.33	1.44	1.67	13.94
**Minimum**	17	9	0.28	1.4	1.6	2	6
**Maximum**	57	88	67.76	6.48	9.7	8	66

## Data Availability

Not applicable.
